# Chorionic-based intrahepatic cholestasis in pregnancy on perinatal outcome in twin pregnancies

**DOI:** 10.1097/MD.0000000000041109

**Published:** 2025-01-10

**Authors:** Na Deng, Yi Liu, Dan Qian, Wei Yi, Han Luo, Diyuan Zhang, Jiajia He

**Affiliations:** a Dianjiang People’s Hospital of Chongqing, Chongqing, China; b Chongqing Medical University, Chongqing, China; c Southwest Medical University, Sichuan, China.

**Keywords:** chorionic, intrahepatic cholestasis in pregnancy, twin pregnancy

## Abstract

This study investigates the impact of twin intrahepatic cholestasis in pregnancy (ICP) in different chorionicity scenarios on pregnancy outcome and risk factors. This retrospective study was designed to investigate the association between ICP and pregnancy outcomes and associated risk factors. Logistic regression analysis was used to verify the correlation between ICP and pregnancy outcome and the associated risk factors with the risk of ICP. Pregnant women with ICP had less gestational weight gain (16.19 ± 5.28 vs 17.78 ± 7.19, *P* = .018), a smaller number of deliveries (16.26% vs 26.40%, *P* = .016), and less spontaneous pregnancy (50.41% vs 61.73%, *P* = .019). The mean birth weight of pregnant women without ICP was lower (2328.07 ± 461.82 vs 2404.70 ± 504.58, *P* = .023), and the prepregnancy hepatitis B virus (HBV) antigen carrying rate was lower (12.20% vs 6.16%, *P* = .021). Pregnancy weight gain (0.95 (0.92, 0.99) *P* = .009) and the number of weeks in labor (0.88 (0.81, 0.96) *P* = .003) were negatively associated with the risk of ICP. Assisted reproductive technology use (1.38 (0.70, 1.79) *P* = .635) and HBV carrier before pregnancy (2.51 (1.42, 4.48) *P* = .002) were positively associated with ICP risk. In monochorionic twins, those with ICP were more likely to have abnormal amniotic fluid (15.79% vs 2.16%, *P* = .012), while those without ICP were less likely to have abnormal amniotic fluid (0.90 (0.83, 0.97) *P* = .004). In double chorion twins, the incidence of preterm birth was higher in women with ICP (40.00% vs 24.14, *P* = .002), and the risk of preterm birth was reduced in women without ICP (0.87 (0.79, 0.96) *P* = .005). In terms of neonatal outcomes, women with ICP were more likely to have a stillbirth (5.26% vs 0.48% *P* = .037), and stillbirth was less likely to occur without ICP (0.95 (0.92, 0.98) *P* = .002). Our study illustrates that twin pregnancies with maternal comorbid ICP have lower birth weight, degree of weight gain during pregnancy and prepregnancy HBV carriage is strongly associated with the development of ICP. ICP contributes to adverse perinatal outcomes such as stillbirth, preterm labor, and differentiates between different chorionic twin pregnancy outcomes. The risk of ICP is differently affected by the degree of weight gain during pregnancy, gestational week of delivery, assisted reproductive technology, and prepregnancy HBV carriage.

## 1. Introduction

With the development of assisted reproductive technology (ART) and the increase of advanced maternal age, the incidence of twin pregnancies has been increasing globally in recent years.^[1,2]^ Dual pregnancies are physiologically and psychologically significantly different from singleton pregnancies and are more prone to preterm labor, low birth weight, intrauterine growth restriction, and other related pregnancy complications compared to singleton pregnancies.^[3]^

Intrahepatic cholestasis in pregnancy (ICP) is a pregnancy-related liver disease that occurs commonly in women.^[4]^ It usually develops in the middle or late stages of pregnancy and is characterized by pruritus and abnormally elevated levels of bile acids and/or alanine aminotransferase. Maternal clinical symptoms resolve rapidly after delivery, but the disease has a high risk of recurrence in subsequent pregnancies. ICP has a global prevalence ranging from 0.5% to 25% in different populations,^[5–7]^ with an incidence of about 1% to 4% in our country, significantly higher risk of occurrence in twin pregnancies than in singleton pregnancies.^[4]^ Studies have also indicated that when ICP is combined with gestational diabetes mellitus (GDM), hypertensive disorders of pregnancy (HDP), and other complications, the incidence of ICP increases.^[8]^ Reasons for the occurrence of ICP are not fully understood and may be related to genetic, hormonal, and environmental factors.^[9]^ While the current study revealed that twin pregnancies combined with ICP adversely affect perinatal outcomes, this effect is more pronounced at the fetal level.^[10]^ And for the time being there are no available studies analyzing it in combination with chorionicity.

With our first aim to assess the risk factors for ICP in twin pregnancies, the second aim was to explore the perinatal outcomes of different chorionic twin pregnancies combined with twin pregnancies.

## 2. Materials and methods

### 2.1. Study design

This study is a retrospective cohort study in the First Affiliated Hospital of Chongqing Medical University, China. We review all electronic medical records of pregnant woman who gave birth to twins during 2015 to 2021. Research for hospital ethics committee approval.

### 2.2. Study populations

A total of 2008 pregnant women who gave birth to twins was hospitalized in the First Affiliated Hospital of Chongqing Medical University, China. The inclusion criteria: (1) gestational age exceeds 28 weeks; (2) regular prenatal examination beginning in 13 weeks in the First Affiliated Hospital of Chongqing Medical University; (3) delivered in the First Affiliated Hospital of Chongqing Medical University. The exclusion criteria: (1) multifetal pregnancy reduction; (2) fetal chromosome abnormality; (3) intrauterine death of fetus; (4) information loss of electronic medical records. Finally, 1398 pregnant women were included in this study to analyze. The research process was conducted anonymously. There is no special informed consent form.

### 2.3. Study variables

All information of pregnancy was extracted from electronic medical records, including: marital age, height, weight, body mass index (BMI), weight gain during pregnancy, gravidity, parity, use of ART, chorionic, delivery gestational age, complications during pregnancy, birthweight, fetus gender, neonatal outcome.

### 2.4. Relative definitions

General information was collected in hospital medical records. Chorionic was confirmed by sonographic examination, including double chorion (DC) and monochorionic (MC).^[11]^ ICP was defined as a total concentration of bile acids exceeding 10 mmol/L, and severe ICP was diagnosed if it exceeded 40 mmol/L.^[12]^ Maternal BMI was calculated by dividing weight (kg) by height (m^2^), including underweight (BMI < 18.5 kg/m^2^), normal weight (18.5–23.99 kg/m^2^), overweight (24–27.99 kg/m^2^), obese (≥28 kg/m^2^).^[13]^ The delivery gestational age was calculated from the date of embryo transfer for in vitro fertilization pregnancies (add 14 days.) and based on the last menstrual period for spontaneous pregnancies, it was confirmed by sonography. Small for gestational age was defined as when the birthweight was below the 10th percentile for gestational age and sex based on twin birthweight curves in Chinese twin.^[14]^ Large for gestational age fetus was indicated infants whose birth weight is above the 90th percentile of mean weight for the same gestational age.^[15]^ Growth discordance was defined as twin birthweight difference < 20%, it was calculated as ((large fetus birthweight - small fetus birthweight)/large fetus birthweight) × 100.^[11]^ HDP was defined as blood pressure ≥ 140/90 mm Hg after 20 weeks of gestational in the absence of proteinuria.^[16]^ GDM was defined as oral 75 g glucose tolerance test between 24 and 28 weeks (fasting plasma glucose ≥ 5.1 mmol/L or 1-hour plasma glucose ≥ 10.0 mmol/L or 2-hour plasma glucose ≥ 8.5 mmol/L).^[17]^

### 2.5. Statistical analysis

All statistical analyses were performed using Stata MP 17.0 (StataCorp, College Station, TX). Continuous variables were expressed as mean (standard deviation) and categorical data were expressed as frequency (percentage). The Shapiro–Wilk W test was used to test the normality of the data. For analysis of variance, Student *t* test or one-way ANOVA was used for normally distributed continuous data and chi-square test was used for normally distributed categorical data. For non-normally distributed data, Mann–Whitney *U* test or Kruskal–Wallis *H* test was used. Logistic regression was used to validate the effect of ICP on perinatal outcomes. Beta coefficients or odds ratios (ORs) and 95% confidence intervals (CIs) were calculated. Adjusted potential confounders included maternal age, prepregnancy BMI, primiparity, mode of conception and chorionicity.

## 3. Results

**Figure 1.**
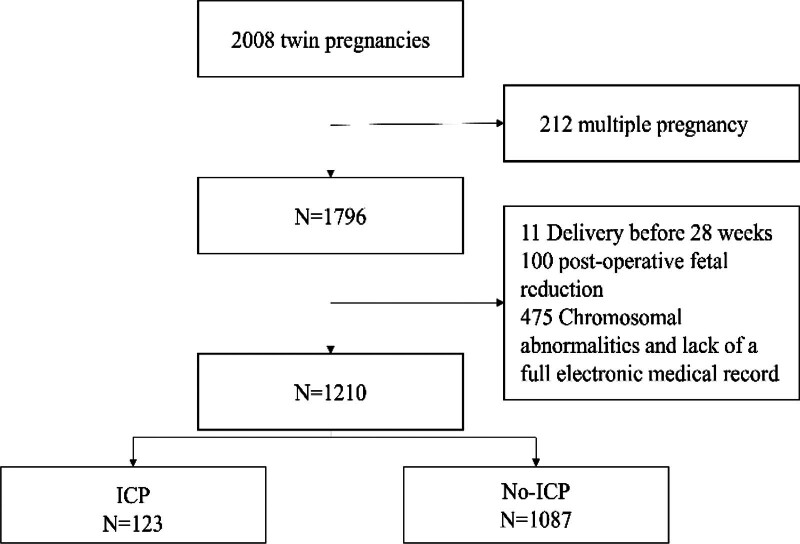
Flowchart of the screening process in this study.

A total of 2008 twin pregnancies were delivered in our hospital from 2015 to 2021 (Fig. 1). In this study, 39 triplet pregnancies, 11 deliveries before 28 weeks, 100 multiple pregnancies after fetal reduction, 648 chromosomal abnormalities, and pregnant women without complete electronic medical records were excluded, 1210 cases were finally included in the study. The flowchart of participant selection for this study is shown in Figure 1. ICP incidence in the present study was 10.17%.

Amongst all 1210 participants, 123 (10.17%) cases were given the diagnosis of ICP, 345 (28.51%) cases were given the diagnosis of GDM, 111 (9.71%) cases were given the diagnosis of HDP, and 21 twin pregnancies with HDP and GDM were diagnosed (1.74%). Grouping of the sample according to diagnosis is specified in Table 1. Based on the results of the analysis, it can be seen that twin pregnant women diagnosed with ICP had lower pregnancy weight gain (16.19 ± 5.28 vs 17.78 ± 7.19 *P* = .018), less number of pregnant women with number of births of 1 (16.26% vs 26.40% *P* = .016), lower incidence of spontaneous pregnancies (50.41% vs 61.73% *P* = .019), lower mean birth weight (2328.07 ± 461.82 vs 2404.70 ± 504.58 *P* = .023), and lower prepregnancy hepatitis B antigen carrier rate in pregnant women with undiagnosed ICP (12.20% vs 6.16% *P* = .021). No statistical differences were found in other outcomes.

**Table 1:** Baseline characteristics of the study subjects.

Variables	Total N = 1210	ICP N = 123	NO-ICP N = 1087	*P*
Age, years	30.22 ± 4.36	30.40 ± 4.22	30.20 ± 4.38	.63
Age category, n (%)				
<25	114 (9.42)	8 (6.50)	106 (9.75)	.33
25–35	909 (75.12)	94 (76.42)	811 (74.61)	.66
≥ 35	187 (15.45)	21 (17.07)	170 (15.64)	.70
Prepregnancy BMI (kg/m^2^)	21.59 ± 2.96	21.50 ± 2.76	21.59 ± 2.99	.74
Underweight, n (%)	154 (12.73)	16 (13.01)	138 (12.70)	.89
Normal weight, n (%)	901 (74.46)	91 (73.98)	810 (74.52)	.91
Overweight, n (%)	143 (11.82)	16 (13.01)	127 (11.68)	.66
Obesity, n (%)	12 (0.99)	0	18 (1.66)	–
Gestational weight gain (kg)	17.61 ± 7.04	16.19 ± 5.28	17.78 ± 7.19	.02
Nulliparity, n (%)	867 (71.65)	97 (78.86)	770 (70.84)	.07
Parity	0.32 ± 0.54	0.27 ± 0.57	0.32 ± 0.54	.28
0	867 (71.65)	97 (78.86)	770 (70.84)	.07
1	307 (25.37)	20 (16.26)	287 (26.40)	.02
2	30 (2.48)	5 (4.07)	25 (2.30)	.22
≥ 3	6 (0.50)	3 (2.44)	5 (0.46)	.15
Spontaneous, n (%)	733 (60.58)	62 (50.41)	671 (61.73)	.02
ART use, n (%)	477 (39.42)	61 (49.59)	416 (38.27)	.02
MC	454 (37.52)	38 (30.89)	416 (38.27)	.12
DC	756 (62.48)	85 (69.11)	671 (61.73)	.12
Cesarean section history, n (%)	182 (15.04)	15 (12.20)	167 (15.36)	.43
HBsAg, n (%)	82 (6.78)	15 (12.20)	67 (6.16)	.02
GDM	345 (28.51)	43 (34.96)	302 (27.78)	.11
HDP	111 (9.71)	11 (8.94)	100 (9.20)	
GH	42 (3.47)	7 (5.69)	35 (3.22)	.18
PE	69 (5.70)	4 (3.25)	65 (5.98)	.30
Hyperthyroidism, n (%)	22 (1.82)	1 (0.81)	21 (1.93)	.72
Hypothyroidism, n (%)	99 (8.18)	16 (13.01)	83 (7.64)	.05
Sex				
Male–female	318 (26.28)	41 (33.33)	277 (25.48)	.07
Female–female	408 (33.72)	42 (34.15)	366 (33.67)	.92
Male–male	484 (40.00)	40 (32.52)	444 (40.85)	.08
Gestational age at delivery, weeks	36.31 ± 9.86	35.56 ± 1.91	36.39 ± 10.38	.38
Birthweight (g)	2396.91 ± 500.85	2328.07 ± 461.82	2404.70 ± 504.58	.02
Larger birthweight (g)	2548.29 ± 468.24	2488.87 ± 421.78	2555.02 ± 472.92	.14
Smaller birthweight (g)	2245.53 ± 486.60	2167.27 ± 445.27	2254.39 ± 490.46	.06
Intertwin weight discordance (g)	302.76 ± 254.44	321.60 ± 257.80	300.63 ± 254.09	.39
Discordant twins, n (%)	214 (17.69)	28 (22.76)	186 (17.11)	.13
Intertwin weight discordance (%)	0.12 ± 0.10	0.13 ± 0.11	0.12 ± 0.10	.22

± represents SD. BMI = body mass index, DC = dichorionic, GDM = gestational diabetes mellitus, HDP = hypertension disorders of pregnancy, ICP = intrahepatic cholestasis in pregnancy, MC = monochorionic.

The impact of different factors on the incidence of twin pregnancies with and without controlling for confounding factors was statistically analyzed (Table 2). It is evident that the degree of weight gain during pregnancy, the gestational week of delivery, the use of ART and prepregnancy hepatitis B virus (HBV) carriage all have an impact on the incidence of ICP. Of these, the latter 2 increased the incidence of ICP, and their effect on the degree of risk of ICP decreased after controlling for confounding factors, but was still statistically different. While the results of the first 2 showed a statistically significant difference in the risk of ICP with increasing degree of weight gain during pregnancy and maternal gestational week of delivery, the adjusted results remained significant. Of note, maternal gestational week increased the risk of ICP after adjustment and to a greater extent than before adjustment, although the results were not statistically significant before adjustment.

**Table 2:** Factors influencing the risk of ICP occurrence.

Variables	Crude OR		*P*	Adjusted OR		*P*
Age	1.01	0.97, 1.05	.63	1.01	0.96–1.06	.84
<25	0.64	0.26, 1.36	.24	0.68	0.40, 1.14	.14
25–35	1.10	0.71, 1.77	.66	1.01	0.93, 1.01	.75
≥ 35	1.11	0.64, 1.85	.68	1.04	0.88, 1.24	.63
Prepregnancy BMI	0.99	0.93, 1.05	.74	0.98	0.92, 1.05	.61
Underweight	0.94	0.51, 1.65	.83	0.97	0.44, 2.14	.93
Normal weight,	0.97	0.63, 1.54	.90	0.97	0.58, 1.11	.67
Overweight	1.13	0.64, 2.00	.67	1.05	0.68, 1.63	.82
Obesity	0	0, 1.82	.15	–	–	–
Gestational weight gain	0.96	0.93, 0.99	.01	0.95	0.92, 0.99	.01
Gestational age at delivery	0.89	0.82, 0.97	.01	0.88	0.81, 0.96	<.01
Birthweight	1.00	1.00, 1.00	.23	1.00	1.00, 1.00	.15
Larger birthweight	1.00	1.00, 1.00	.14	1.00	1.00, 1.00	.07
Smaller birthweight	1.00	1.00, 1.00	.06	1.00	1.00, 1.00	.03
Parity	1.54	0.97, 2.52	.06	2.24	1.04, 4.85	.04
Nulliparity	1.54	0.97, 2.52	.06	1.34	0.80, 2.24	.27
ART use	1.58	1.07, 2.35	.02	1.38	0.70, 1.79	.64
Cesarean section history	0.76	0.40, 1.36	.35	1.24	0.55, 2.81	.60
HBsAg	2.11	1.08, 3.90	.01	2.51	1.42, 4.48	<.01

Adjust: age, BMI, nulliparity, method of conception, chorionic; BMI = body mass index, CI = confidence interval, HBsAg = hepatitis B surface antigen, OR = odds ratio.

To analyze the perinatal outcomes of mothers and infants due to ICP, we stratified them by chorionicity (Tables 3 and 4). It is observed that in MC twin pregnancies, ICP pregnant women were more likely to have amniotic fluid abnormalities (15.79% vs 2.16% *P* = .012), whereas the absence of a diagnosis of ICP reduced the risk of amniotic fluid abnormalities (0.90 (0.83, 0.97) *P* = .004). In neonatal outcomes, pregnant women with ICP had a higher likelihood of stillbirth (5.26% vs 0.48% *P* = .037), and the absence of combined ICP reduced the risk of stillbirth (0.95 (0.92, 0.98) *P* = .002). Rest of the results suggest that combining ICP increases the risk of adverse perinatal outcomes for both mother and baby, although the results were not statistically different.

**Table 3:** Effect of ICP on perinatal outcomes in MC twins.

Variables	ICP	No-ICP	*P*	OR	*P*	AOR (95%Cl)	*P*
Maternal outcomes							
Cesarean section, n (%)	37 (97.37)	398 (95.67)	1.00	1.02	.635	1.02 (0.95, 1.09)	.63
Preterm birth, n (%)	8 (21.05)	78 (18.75)	.67	0.92	.310	0.92 (0.78, 1.08)	.32
Postnatal hemorrhage, n (%)	2 (5.26)	16 (3.85)	.66	0.99	.669	0.99 (0.92, 1.05)	.66
Amniotic fluid abnormality, n (%)	6 (15.79)	19 (2.16)	.01	0.89	.004	0.90 (0.83, 0.97)	.01
Neonatal outcomes							
Stillbirth, n (%)	2 (5.26)	2 (0.48)	.04	0.95	.002	0.95 (0.92, 0.98)	.01
LGA, n (%)	8 (21.05)	77 (18.51)	1.00	0.97	.701	0.97 (0.85, 1.10)	.66
SGA, n (%)	7 (18.42)	56 (13.46)	.60	0.95	.378	0.96 (0.85, 1.07)	.44
Fetal distress, n (%)	3 (7.89)	49 (11.78)	.60	1.00	Omitted		
Fetal asphyxia, n (%)	0 (0.00)	7 (1.68)	1.00	1.02	.421	1.02 (0.98, 1.06)	.38
NICU, n (%)	10 (26.32)	102 (24.52)	.84	0.98	.826	0.98 (0.85, 1.23)	.77

Adjust: age, BMI, nulliparity, method of conception. AOR = adjusted odds ratio, CI = confidence interval, LGA = large for gestational age fetus, NICU = Neonatal Intensive Care Unit, OR = odds ratio, SGA = small for gestational age.

**Table 4:** Effect of ICP on perinatal outcomes in DC twins.

Variables	ICP	No-ICP	*P*	OR	*P*	AOR (95%Cl)	*P*
Maternal outcomes							
Cesarean section, n (%)	82 (96.47)	658 (98.06)	.41	0.99	.425	0.98 (0.95, 1.02)	.32
Pretermbirth, n (%)	34 (40.00)	162 (24.14)	.01	0.87	.004	0.87 (0.79, 0.96)	.01
Postnatal hemorrhage, n (%)	3 (3.53)	29 (4.32)	1.00	1.01	.773	1.01 (0.77, 1.06)	.58
Amniotic fluid abnormality, n (%)	6 (7.06)	25 (3.73)	.15	0.97	.145	0.97 (0.92, 1.01)	.15
Neonatal outcomes							
Stillbirth, n (%)	0 (0.00)	0 (0.45)	1.00	1.00	.537	1.00 (0.98, 1.02)	.56
LGA, n (%)	25 (29.41)	205 (30.55)	.90	1.01	.830	1.01 (0.91, 1.12)	.83
SGA, n (%)	4 (4.71)	32 (4.77)	1.00	1.00	.979	1.00 (0.95, 1.12)	.94
Fetal distress, n (%)	1 (1.18)	43 (6.41)	.05	1.00	Omitted		
Fetal asphyxia, n (%)	0 (0.00)	1 (0.15)	1.00	1.00	.722	1.00 (0.99, 1.01)	.96
NICU, n (%)	14 (16.47)	88 (13.11)	.40	0.97	.459	0.98 (0.90, 1.06)	.55

Adjust: age, BMI, nulliparity, method of conception. AOR = adjusted odds ratio, CI = confidence interval, LGA = large for gestational age fetus, NICU = Neonatal Intensive Care Unit, OR = odds ratio, SGA = small for gestational age.

Preterm labor incidence was higher in pregnancies with combined ICP (40.00% vs 24.14 *P* = .002) and not combining ICP reduced the risk of preterm labor (0.87 (0.79, 0.96) *P* = .005) in twin pregnancies with DC villus. None of the remaining outcomes were statistically different, but consistent with MC twins, combined ICP increases the risk of adverse maternal and infant perinatal outcomes, but it appears to be to a lesser extent in DC twins.

## 4. Discussion

Results of the above study showed that the incidence of GDM was also higher than the average in China (28.51% vs 12.97%), and the incidence of HDP was comparable to the average (9.71% vs 9.95%), which may be due to the difference in dietary habits and lifestyles.^[18]^ ICP prevalence was significantly different from our average (10.17% vs 1–4%), which may be related to the increased risk of pregnancy complications due to twin pregnancies.^[3,19]^ The fact that our study reveals that prepregnancy HBV carriage increases the risk of developing ICP is consistent with previous findings.^[20–25]^ Stratified analyses indicated that ICP adversely affects maternal and infant perinatal outcomes, in MC twins it leads to an increased risk of amniotic fluid abnormalities as well as stillbirth, and in DC twins it leads to an increased risk of preterm labor, and the rest of the results was not statistically different, which is in line with previous studies that increased the risk of perinatal outcomes. The risk of adverse perinatal outcomes varied among chorionicity in our study, which may be related to differences in pathophysiologic conditions of twin fetuses with different chorionicity. ICP’s most serious perinatal outcome is stillbirth with a high risk of stillbirth, which may be related to acute vasoconstriction of placental chorioallantoic vessels and umbilical vein vessels due to elevated bile acids in the body,^[26,27]^ but not to chronic placental insufficiency. Animal studies have also shown that taurocholic acid induces loss of cardiomyocyte beating in rats, which may also correlate with stillbirth.^[28]^ While our study suggested a higher risk of stillbirth due to ICP in MC twins, it was not reflected in DC twins, and we speculate that this may be related to the different placental structures of MC and DC. Elevated levels of bile acids have been shown to promote myometrial contractions and increase oxytocin bioactivity may correlate with preterm labor occurring in ICP.^[26,29–32]^ However, this still does not explain the differential results they produce in different chorionic twin fetuses.

Further analysis of the study showed that prepregnancy HBV carriage and the use of ART increased the risk of ICP, whereas the degree of weight gain during pregnancy and the gestational week of delivery were negatively associated with the risk of ICP. This finding has not yet been described in the other literature. Although the underlying mechanism is not clear, we speculate that it may be related to hepatocellular damage induced by the replicative state of the virus in vivo, causing a systemic inflammatory response in hepatocytes, which ultimately leads to the deterioration of liver function and an increase in the risk of ICP.^[33,34]^ This may also be related to estrogen disruption due to HBV infection.^[32,35]^ ART is a risk factor for ICP and our findings are in line with previous studies,^[36–39]^ but the underlying mechanisms are not yet clear, although some studies have suggested that twin pregnancies with ART may have earlier and more severe ICP than natural pregnancies, which may be related to ICP induced by hormonal factors such as estrogen and high levels of umbilical cord luteinizing hormone.^[26]^ Another study suggests that abnormal microenvironment and inflammation, as well as metabolic factors during implantation and pregnancy, may be associated with a higher risk of ICP.^[40]^

No literature exists on the effect of weight gain during pregnancy on ICP, our study suggests that the degree of weight gain during pregnancy is negatively correlated with the risk of developing ICP, but weight management during pregnancy has been correlated with the development of many pregnancy complications, and more research is needed to elucidate the relationship between weight gain during pregnancy and ICP. ICP risk was negatively correlated with gestational week of delivery, reflecting the fact that the higher the risk, the earlier the gestational week of delivery for twin pregnancies with ICP, and another singleton clinical study showed that the risk of expectant treatment in ICP pregnancies was higher after maturation of the fetal lungs than the risk of delivery, and that the risk of expectant treatment continued to increase beyond 36 weeks.^[41]^

Besides, our findings did not indicate that ICP was correlated with GDM and HDP, probably due to the low incidence of ICP and its small sample size, which needs to be further expanded for the study.

Overall, our study illustrates that twin pregnancies with maternal comorbid ICP have lower birth weight, degree of weight gain during pregnancy and prepregnancy HBV carriage is strongly associated with the development of ICP. ICP contributes to adverse perinatal outcomes such as stillbirth, preterm labor, and differentiates between different chorionic twin pregnancy outcomes. The risk of ICP is differently affected by the degree of weight gain during pregnancy, gestational week of delivery, ART, and prepregnancy HBV carriage.

At last, this study hopes to provide new clinical evidence for the study of ICP in twin pregnancies combined with ICP.

Advantages of our study include the fact that we present for the first time the effect of weight gain during pregnancy on the risk of ICP. Second, we stratified the sample size for chorionicity to analyze the effect of ICP on perinatal outcomes in mothers and infants. Finally, the sample size of the study was relatively large and it illustrates our conclusions to some extent.

Yet, there are some limitations to this study. First, the sample was obtained from a single center, which may have a medical selection bias and cannot be shown to be regionally representative. Second, our study is only a clinical study, the causes of which have not been thoroughly investigated, and more research is needed to reveal the underlying molecular mechanisms.

## Author contributions

**Conceptualization:** Jiajia He, Na Deng, Yi Liu, Wei Yi.

**Data curation:** Jiajia He, Na Deng.

**Investigation:** Jiajia He, Na Deng, Yi Liu, Dan Qian, Han Luo, Diyuan Zhang.

**Methodology:** Jiajia He, Yi Liu, Dan Qian, Wei Yi, Han Luo, Diyuan Zhang.

**Project administration:** Jiajia He.

**Resources:** Na Deng.

**Supervision:** Jiajia He.

**Visualization:** Na Deng, Yi Liu, Dan Qian, Han Luo.

**Writing – original draft:** Jiajia He, Na Deng, Yi Liu, Han Luo.

**Writing – review & editing:** Jiajia He, Na Deng.
